# Randomized comparison of observation unit plus stress cardiac MRI and hospital admission

**DOI:** 10.1186/1532-429X-11-S1-O103

**Published:** 2009-01-28

**Authors:** Chadwick D Miller, Wenke Hwang, James W Hoekstra, Cedric Lefebvre, Doug Case, W Gregory Hundley

**Affiliations:** Wake Forest University School of Medicine, Winston-Salem, NC USA

**Keywords:** Acute Coronary Syndrome, Intermediate Risk, Index Hospitalization, Cardiac Marker, Observation Unit

## Introduction

Adoption of an observation unit (OU) strategy in patients with chest pain at intermediate-risk for ACS has been hampered by limitations of traditional cardiac testing. As a result, most intermediate risk patients are admitted to the hospital for their evaluation. CMR demonstrates superior accuracy compared to other testing modalities. Additionally, CMR is with highly sensitive to recent or ongoing infarction which may allow stress imaging to be performed without waiting for the results of serial cardiac markers. These advantages of CMR make it well suited for use in OUs. An OU-CMR strategy may be resource saving compared to hospital admission.

## Purpose

To compare resource consumption between an observation unit stress cardiac MRI (OU-CMR) strategy and hospital admission when used to evaluate emergency department (ED) patients with chest pain at intermediate risk for acute coronary syndrome (ACS).

## Methods

Patients meeting intermediate risk criteria (TIMI risk score ≥ 2 or clinical impression of intermediate risk) underwent stratified blocked randomization to OU-CMR or hospital admission. OU-CMR participants underwent cardiac markers at 0, 4, and 8 hours with adenosine or dobutamine CMR imaging performed at the first available time after the return of the first two cardiac marker results. CMR imaging included resting wall motion, T2 weighted imaging, and perfusion, stress wall motion and perfusion, and delayed enhancement. Hospital admission participants underwent evaluations as determined by their treating physician. Participants were contacted at 30 days to determine events occurring after hospital discharge. Primary outcomes included direct cost of the index hospitalization and length of stay. Cost was calculated using cost:charge ratios and physician work-RVUs. The results of the first 50 participants are reported in this interim analysis.

## Results

Participants had a mean age of 56 years, a median TIMI risk score of 2 (Q1 = 2, Q3 = 3), 25 (50%) were female, and 10 (20%) reported prior coronary disease. Participants were equally randomized with 25 participants in each treatment group. Protocol adherence was high among both groups (24/25 [96%]OU-CMR, 23/25 [92%] standard care remained until hospital discharge). Stress CMR imaging was obtained in 24/25 (96%) OU-CMR participants during the index visit; 11/25 (44%) received imaging the same day as presentation. In the OU-CMR group, 20/25 (80%) of participants were discharged from the OU without admission. Five participants were admitted from the OU and underwent continued monitoring (n = 1) or cardiac catheterization (n = 4). Of the 4 undergoing cardiac catheterization, 1 was diagnosed with a non-ischemic emergent cardiac condition and 3 were not found to have an emergent cause of chest pain. There were no complications related to CMR testing. Among standard care participants, all patients were admitted, 21/25 (84%) had cardiac imaging with 14 undergoing stress echocardiography, 3 stress cardiac MRI, 3 cardiac catheterizations, and 1 resting echo exam.

ACS criteria were met in 3/50 (6%) participants due to revascularization (n = 2) and myocardial infarction (n = 1), all in the hospital admission arm during the index hospitalization. Two participants had repeat hospitalizations for chest pain within 30 days, both in the hospital admission arm, and neither met ACS criteria. The OU-CMR group had a trend towards same day discharge more frequently than standard care participants (7/25 (28%) vs 3/25 (12%), p = 0.29). Cost of index hospitalization demonstrated a near significant trend towards favoring OU-CMR before adjustment (mean $2823 vs $4342, p = 0.12) and after adjustment for covariates ($1537 difference, p = 0.10). Length of stay demonstrated a trend towards favoring OU-CMR before (mean 27.5 h vs 31.4 h, p = 0.38) and after adjustment (5.5 h difference, p = 0.24).

## Conclusion

An OU-CMR approach among ED patients with chest pain at intermediate risk for ACS is feasible. OU-CMR decreases hospital admissions and has demonstrated a strong near-significant trend towards decreasing index hospitalization cost.

